# Early bruising detection of ‘Korla’ pears by low-cost visible-LED structured-illumination reflectance imaging and feature-based classification models

**DOI:** 10.3389/fpls.2023.1324152

**Published:** 2023-11-16

**Authors:** Mengwen Mei, Zhonglei Cai, Xinran Zhang, Chanjun Sun, Junyi Zhang, Huijie Peng, Jiangbo Li, Ruiyao Shi, Wei Zhang

**Affiliations:** ^1^ College of Mechanical and Electrical Engineering, Shihezi University, Shihezi, China; ^2^ Jiangsu Province and Education Ministry Co-sponsored Synergistic Innovation Center of Modern Agricultural Equipment, Jiangsu University, Zhenjiang, China; ^3^ Xinjiang Production and Construction Corps Key Laboratory of Modern Agricultural Machinery, Shihezi, China; ^4^ Engineering Research Center for Production Mechanization of OasisCharacteristic Cash Crop, Ministry of Education, Shihezi, China; ^5^ Intelligent Equipment Research Center, Beijing Academy of Agriculture and Forestry Sciences, Beijing, China; ^6^ National Engineering Research Center for Information Technology in Agriculture, Beijing, China; ^7^ Department of Computer Technology and Science, Anhui University of Finance and Economics, Bengbu, China

**Keywords:** pears, early bruise detection, classification, machine learning, visible LED structured illumination

## Abstract

**Introduction:**

Nondestructive detection of thin-skinned fruit bruising is one of the main challenges in the automated grading of post-harvest fruit. The structured-illumination reflectance imaging (SIRI) is an emerging optical technique with the potential for detection of bruises.

**Methods:**

This study presented the pioneering application of low-cost visible-LED SIRI for detecting early subcutaneous bruises in ‘Korla’ pears. Three types of bruising degrees (mild, moderate and severe) and ten sets of spatial frequencies (50, 100, 150, 200, 250, 300, 350, 400, 450 and 500 cycles m^-1^) were analyzed. By evaluation of contrast index (CI) values, 150 cycles m^-1^ was determined as the optimal spatial frequency. The sinusoidal pattern images were demodulated to get the DC, AC, and RT images without any stripe information. Based on AC and RT images, texture features were extracted and the LS-SVM, PLS-DA and KNN classification models combined the optimized features were developed for the detection of ‘Korla’ pears with varying degrees of bruising.

**Results and discussion:**

It was found that RT images consistently outperformed AC images regardless of type of model, and LS-SVM model exhibited the highest detection accuracy and stability. Across mild, moderate, severe and mixed bruises, the LS-SVM model with RT images achieved classification accuracies of 98.6%, 98.9%, 98.5%, and 98.8%, respectively. This study showed that visible-LED SIRI technique could effectively detect early bruising of ‘Korla’ pears, providing a valuable reference for using low-cost visible LED SIRI to detect fruit damage.

## 1 Introduction

Bruising is the most common type of mechanical damage (Opara and Pathare, 2014), particularly on fruit like pears which are sensitive to mechanical damage (Celik, 2017). Bruises may occur when the stress on the fruit surface exceeds the failure stress of fruit tissue. It is a kind of subcutaneous tissue injury without rupture of the skin of fruit (Opara and Pathare, 2014; Hussein et al., 2019; Mei and Li, 2023). The formation of bruise will not only lead to physiological changes in fruit density, moisture content, browning degree and firmness, but also accelerate the respiration rate of fruit and increase the production of ethylene (Khurnpoon and Siriphanich, 2011; Polat et al., 2012; Bian et al., 2020), thereby accelerating the decay process of fruit and leading to significant economic losses. However, the non-destructive and accurate detection of early bruised fruit is extremely challenging.

Many techniques have been used for bruising detection of pears, including hyperspectral imaging (HSI) (Lee et al., 2014; Fu and Wang, 2022; Tian et al., 2023), magnetic resonance imaging (MRI) (Razavi et al., 2018; Razavi et al., 2020), X-ray computed tomography (CT) (Azadbakht et al., 2019a; Azadbakht et al., 2019b), thermal imaging (TI) (Kim et al., 2014; Zeng et al., 2020), Optical coherence tomography (OCT) (Zhou et al., 2019), etc. HSI has been widely used in fruit damage detection and has been proven effective in this regard. However, its capabilities for detection of early-stage bruises, especially immediate post-bruise detection, still require enhancement. Additionally, HSI is too slow and expensive for commercial applications (Tian et al., 2021). For MRI, CT, OCT, they can capture high contrast images but equipment cost is an important consideration factor in practical applications. TI is a detection technology that does not require a light source. It can non-invasively convert the radiation of an object into a surface temperature distribution for bruising detection (Zeng et al., 2020). However, it has strict temperature requirements, and the fruit may be affected by the heating/cooling process.

Traditional imaging systems (e.g. HSI, multispectral imaging and machine vision) commonly used uniform or diffuse illumination for fruit quality detection, making it difficult to control light penetration and interaction with biological tissue, which limits their performance in detecting depth-specific information such as subsurface tissue bruising in fruit (Lu and Lu, 2017; Lu and Lu, 2019). Structured illumination (SI) can be used to enhance the detection of subsurface defects in fruit by varying the spatial frequency of the illumination to control the depth of light penetration into the tissue (Li et al., 2023; Li et al., 2024). Depending on the purpose of the application, SI techniques can be implemented using either inverse or forward methods. Spatial frequency domain imaging (SFDI) based on inverse methods can be used to obtain absorption coefficients and approximate scattering coefficients of fruit tissue by means of inverse algorithmic diffusion models (Sun et al., 2019). This method has also been used for bruise detection in pears (He et al., 2018). Different from SFDI, structured-illumination reflection imaging (SIRI) is used to enhance the detection of subsurface damage of fruit in a simpler and faster way. The pattern image obtained by demodulation can obtain direct component (DC) and alternating component (AC) images, in which the AC image carries depth resolution information and can be used for the detection of subsurface tissue bruising in fruit (Lu et al., 2016a; Li et al., 2023). SIRI has now been used to detect bruises on apples (Lu et al., 2016a) and pickling cucumbers (Lu et al., 2021) with good results. Recently, our laboratory developed a new SIRI system based on light-emitting diode (LED) light source and monochromatic camera, which can realize fruit detection in visible light band, and further reduce the cost of SIRI system while obtaining good subcutaneous damage detection effect. The system has been used to detect the early decay of oranges (Cai et al., 2022).

The aim of this study was to demonstrate the ability of low-cost visible-LED SIRI to detect pear bruising at an early stage. The specific objectives were to: (1) Acquire DC and AC images for ‘Korla’ pears with three types of bruising degrees at ten sets of spatial frequencies using a visible-LED SIRI system to determine the optimal spatial frequency combined with a three-phase image demodulation scheme and contrast index analysis; (2) Extract the texture features of AC and ratio (RT) images through the gray level co-occurrence matrix (GLCM) and select the appropriate features based on the random frog algorithm; (3) Develop the least squares support vector machine (LS-SVM), partial least squares discriminant analysis (PLS-DA), and K-nearest neighbor (KNN) classification models combined with selected texture features to classify sound and bruised pears; and (4) Evaluate the independent bruising degree prediction models and mixed bruising degree prediction model to determine the optimal one for classification of bruised ‘Korla’ pears.

## 2 Materials and methods

### 2.1 Sample preparation

‘Korla’ pears were used in the study. ‘Korla’ pear is a characteristic fruit in Xinjiang, China. It is famous for its fine flesh, juicy juice and strong aroma. However, the peel of this pear is very thin and easily damaged. The ‘Korla’ pears were purchased from a local fruit store in Beijing, China. The ‘Korla’ pear can be roughly divided into two distinct maturation periods, namely, the green maturation period and the yellow maturation period. During the green maturation period, the skin of the pear appears green, while in the yellow maturation period, it turns fully yellow. Over the course of storage, the color of the pear peel undergoes a transition from green to yellow. Most of the pears sold in the fruit store are in the green maturation period, but according to the different sales time, the epidermis of some pears will gradually become yellow, even full yellow, and some pears also may be reddish in color. In this study, the color of the pears was not taken into account during the purchasing process. For all pear samples, green samples accounted for the majority, with a small amount of red or yellow samples. By a simple visual inspection, 403 pears (three pears were used for spatial frequency selection) without external defects were selected as experimental samples. The pear size varies among them. To replicate the real detection environment, this experiment deliberately refrains from making any distinctions.

**Figure 1:**
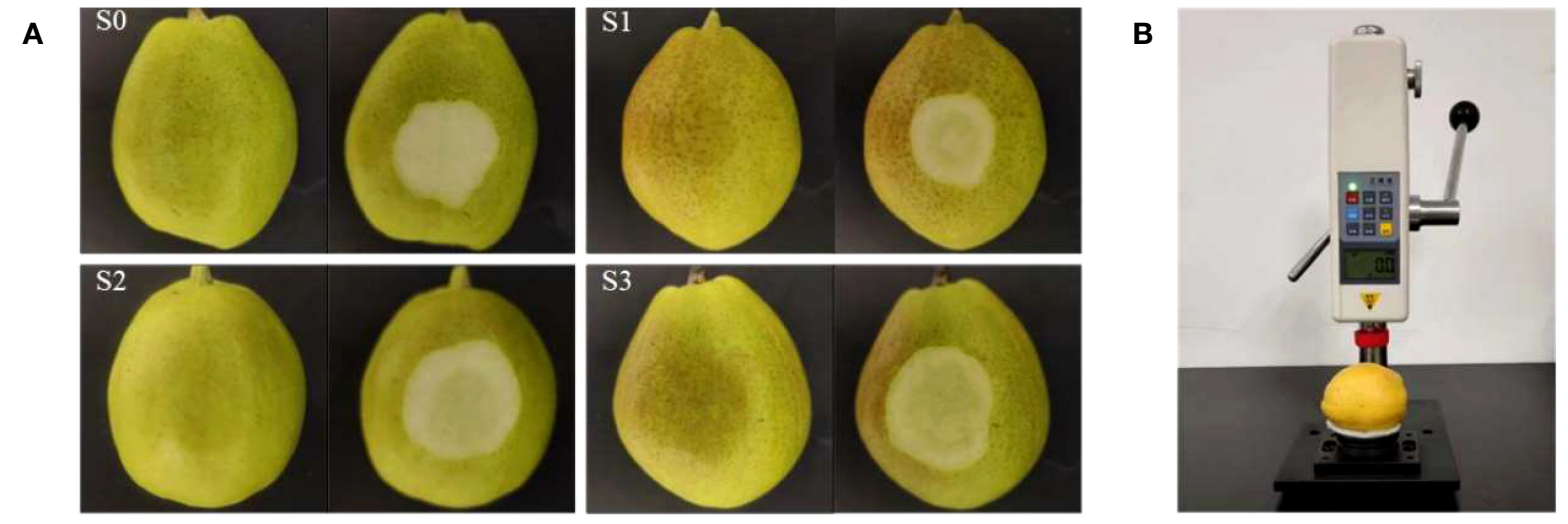
**(A)** Typical ‘Korla’ pear samples (Unpeeled and peeled) with different degrees of bruising (S0: sound, S1: mild bruises, S2: moderate bruises, S3: severe bruises). **(B)** Preparation of bruise samples.

The static load range of pear fruit during harvesting, storage and transportation is 60-200 newtons (N) (Wu et al., 2013). Therefore, this study selected 50 N, 100 N, and 150 N as the static load pressure level to induce early bruising in pears. Four hundred pears were randomly divided into 4 groups, with 100 in each group, which were sound group (recorded as S0), mild bruise group (recorded as S1), moderate bruise group (recorded as S2) and severe bruise group (recorded as S3). The pears were balanced at room temperature (temperature 24°C, humidity 42%) for 24 h. After that, the 100 pears in the S0 group were not treated. The pears of S1, S2 and S3 were bruised by pressing presses. The pressure probe end of the press is a cylindrical plastic with a diameter of 3 cm and is connected to a pressure sensor with a display screen. Due to the high curvature radius of the equatorial part of ‘Korla’ pear, it is more vulnerable to form bruises during sorting and packaging. Consequently, the equatorial section of the pear is chosen and subjected to pressure using a press to induce a static pressure bruise. During sample preparation, the pear sample was placed horizontally under the pressure probe of the press. The pear was placed on a sponge-buffered fruit tray and the handle was slowly pressed. When the pressure sensor display reached a specific reading (50N, 100N and 150N represent S1, S2 and S3, respectively), the pressing was stopped. After standing still for 3 seconds, the handle was slowly loosened and the sample was taken out. **Figure 1A** depicts the RGB images of pears exhibiting three distinct bruise degrees (S1, S2, S3), which also includes the control group (S0). **Figure 1B** shows the preparation of bruise samples.

### 2.2 SIRI system and image acquisition

**Figure 2:**
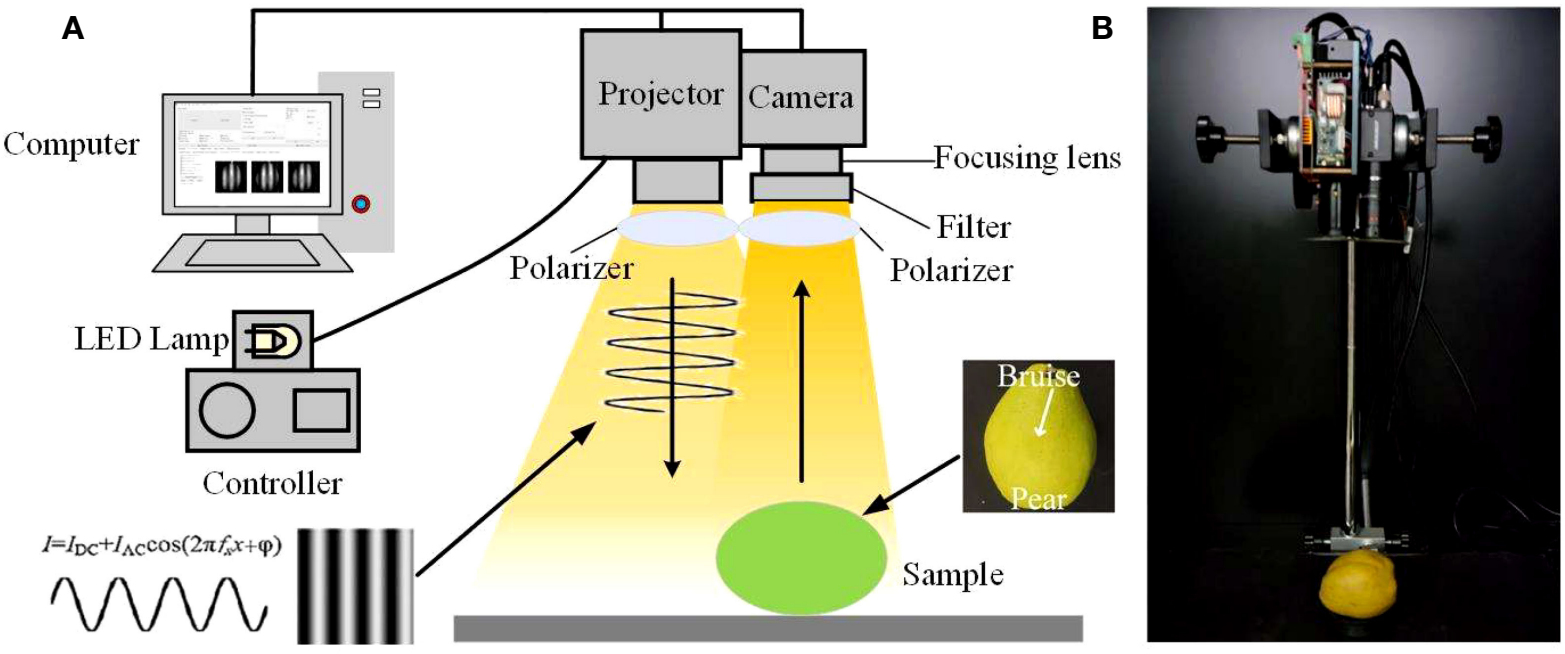
Schematic diagram **(A)** and real system **(B)** of the SIRI system.

The SIRI system used in this experiment is mainly composed of a digital projector (DLP4500, Texas Instruments, Dallas, TX, United States) with visible LED lights, a monochromatic camera (MV-CA050-10GM, Hangzhou Hikrobot Intelligent Technology Co., Ltd., Hangzhou, China) with an adjustable focal length lens (MVL-MF1628M-8MP, Hangzhou Hikrobot Intelligent Technology Co., Ltd., Hangzhou, China), two polarizers (PL-D50, RAYAN Technology Co., Ltd., Changchun, China), a long-wave pass filter (the cut-off frequency is 450 nm) (GCC-300201, Daheng New Epoch Technology Inc., Beijing, China), an adjustable sample stage (600LW-WT, Shanghai Weimu Automation Equipment Co., Ltd., Shanghai, China) and a computer that can perform sampling and data processing (Cai et al., 2022). The projector and the camera are located directly above the sample to be tested, perpendicular to the horizontal axis. Additionally, a pair of linear polarizers is placed in front of the projector and the camera to eliminate specular reflection. The projector and the camera are connected to the computer through the data line and controlled by the computer through the binding software. The basic composition and real system of the SIRI system is shown in **Figure 2**. The SIRI system based on LED light and monochrome camera can obtain SI images in the visible light band, which further reduces the equipment cost.

Images were collected immediately after static pressure was applied to each pear. The sample is positioned on the imaging stage with the bruising area facing upward toward the projector and camera. The height of the platform is adjusted to accommodate all sizes of pears before imaging each sample. The distances from the pear sample to the projector and camera was set at approximately 30 cm. Three phase-shifted sinusoidal patterns (with phase offsets of -2π/3, 0 and 2π/3) in 8-bit bmp format were created in Matlab (The Mathworks, Inc., Natick, MA, USA) and uploaded to the projector control software on the computer, and then imported into the projector for sample illumination. The camera is set to an exposure time of 50 ms to obtain an 8-bit grayscale image for each pattern projected onto the sample.

### 2.3 Image demodulation and processing

The pear pattern image collected from the SIRI system cannot be directly used for bruising detection, but needs further image demodulation processing. The image demodulation method used in this experiment is a three-phase demodulation (TPD) method. It is a commonly used sinusoidal image demodulation scheme. This method requires three images with equal phase steps for image demodulation. Through the SIRI system, three images are obtained at each frequency, and the phase offsets are −2π/3 , 0 and 2π/3 , respectively (Schreiber and Bruning, 2007). Typically, a two-dimensional sinusoidal fringe pattern can be represented as follows:


(1) $$\begin{array}{c} I_{n}\left(x,y\right)=I_{DC}\left(x,y\right)+I_{AC}\left(x,y\right)\cos\left(2\pi f_{x}+2\pi f_{y}+\varphi_{n}\right) \end{array}$$

where $I_{DC}\left(x,y\right)$ and $I_{AC}\left(x,y\right)$ are DC and AC, respectively. $f_{x}$ and $f_{y}$ are the spatial frequencies along the x and y axes, respectively. According to the experimental requirements, only the spatial frequency in one direction is required, so $f_{y}$ is 0 in this experiment. $\varphi_{n}$ is the phase shift of the *n*th pattern image. In this experiment, $\varphi_{1}$ , $\varphi_{2}$ and $\varphi_{3}$ corresponding to the sinusoidal fringe patterns $I_{1}\left(x,y\right)$ , $I_{2}\left(x,y\right)$ and $I_{3}\left(x,y\right)$ are −2π/3 , 0 and 2π/3 , respectively. The DC and AC are the final results obtained by image demodulation, which can be obtained by the following equation (for simplicity, the coordinate symbol is omitted).


(2) $$\begin{array}{c} I_{DC}=\;\frac{1}{3}\left(I_{1}+I_{2}+I_{3}\right) \end{array}$$

(3) $$\begin{array}{c} I_{AC}=\frac{\sqrt{2}}{3}\sqrt{{\left(I_{1}-I_{2}\right)}^{2}+{\left(I_{1}-I_{3}\right)}^{2}+{\left(I_{2}-I_{3}\right)}^{2}} \end{array}$$

The aforementioned equations (3) demonstrate that TPD exclusively relies on straightforward pixel-by-pixel algebraic operations, resulting in efficient computation. Moreover, the subtraction operation effectively mitigates common noise across the three images, enhancing its robustness. The demodulated image DC and AC images correspond to the images acquired under uniform diffuse illumination and the images resulted from the sinusoidal illumination pattern, respectively. The AC image contains depth information, which varies with the spatial frequency of the illumination pattern. Specifically, as the spatial frequency of the illumination patterns increased, the depth of tissue interrogation in the AC images decreased (Lu and Lu, 2019).

Although AC image has the ability of enhanced detection, there are still some deficiencies, such as low intensity, uneven brightness distribution, and large noise. Since DC images also have similar problems, the AC image can be divided by the corresponding DC image to obtain a ratio image RT image to improve the image quality. RT image can make the image background more uniform and enhance the image contrast. It is defined as follows:


(4) $$\begin{array}{c} RT=\frac{I_{AC}}{I_{DC}}=\frac{\sqrt{2}}{I_{1}+I_{2}+I_{3}}\sqrt{{\left(I_{1}-I_{2}\right)}^{2}+{\left(I_{1}-I_{3}\right)}^{2}+{\left(I_{2}-I_{3}\right)}^{2}} \end{array}$$

### 2.4 Spatial frequency selection

Since the different penetration depths of structured light at different spatial frequencies, it is crucial to select the appropriate frequency for accurate detection of pear bruises. Through preliminary experiments, it was found that the detection effect of bruising was good when the spatial frequency was between 0 and 500 cycles m^-1^. Therefore, the spatial frequencies of 50, 100, 150, 200, 250, 300, 350, 400, 450 and 500 cycles m^-1^ were selected for imaging, and the optimal frequency that can accurately detect the bruises was selected by comparing the demodulation results. Prior to conducting the experiment, it is essential to generate sinusoidal fringe patterns with varying spatial frequencies on the computer. The generation formula is presented in equation (1). The value $I_{DC}$ and $I_{AC}$ were set to (255/2). By adjusting the parameters $f_{x}$ or $f_{y}$ within the equation, fringe patterns corresponding to different spatial frequencies can be generated. These fringe patterns are visually recognizable, appearing as densely-packed black and white stripes at higher spatial frequencies, and sparser black and white stripes at lower spatial frequencies.

The contrast index (CI) is introduced to compare the enhancement effect of pear bruises at each spatial frequency. CI can quantitatively evaluate the image contrast, that is, the distinguishability of the bruised part relative to the whole part of the fruit. It needs to divide the pear to be detected into two parts, namely bruised tissue and sound tissue. Afterward, the ratio of the between-class variance to the total variance of the pixel intensity is calculated to obtain CI:


(5) $$\begin{array}{c} CI=\frac{N_{x}{\left(\bar{x}-\bar{z}\right)}^{2}+N_{y}{\left(\bar{y}-\bar{z}\right)}^{2}}{\sum_{i=1}^{N_{z}}{\left(z_{i}-\bar{z}\right)}^{2}} \end{array}$$

where $N_{x}$ , $N_{y}$ , $N_{z}$ are the number of pixels in the bruised, sound tissue and the whole region, respectively. And $\bar{x}$ , $\bar{y}$ and $\bar{z}$ are the average strength of the bruised, sound tissue and the whole region, respectively. The value of CI is between 0 and 1, where a higher value indicates the better visibility and distinguishability of the bruised area.

Calculating the CI involves segmenting both bruised and sound areas, which can be challenging to achieve in AC images depicting mild bruises. On the contrary, RT images are more easily segmented due to contrast enhancement. Consequently, this study opts to employ RT images rather than AC images to calculate CI for optimizing the spatial frequency. After removing the background by threshold segmentation, the Otsu threshold segmentation method (Otsu, 1979) was used to segment the bruise area to obtain the images of the bruised area, the sound area and the whole fruit area, and then the CI value was calculated according to Equation (5). The CI values under different spatial frequencies and different degrees of bruising were compared, and the optimal spatial frequency suitable for all degrees of bruising was selected for the next study.

### 2.5 Feature extraction and selection

Before using the machine learning algorithm to classify the images of pears, it is usually necessary to extract the features of the images, and use the extracted discriminant features to represent the images. Texture is one of the important features used to identify the object or region of interest in the image. Therefore, the texture features are also often applied to image classification in the fruit defect detection (Lu et al., 2021; Cai et al., 2022). Gray level co-occurrence matrix (GLCM) is a commonly used statistical method for image processing and texture analysis. It characterizes the texture of the image by calculating the frequency of pixel pairs with specific values and specific spatial relationships in the image to obtain GLCM, and then extracts statistical measures from the matrix. The Haralick features calculated based on GLCM are functions of distance and angle. In this study, 56 Haralick features with a distance of 1 were extracted in four directions (angles 0°, 45°, 90°135°) (Haralick et al., 1973). Therefore, 56 complete feature sets were extracted from each picture for bruise detection.

Feature selection is the process of selecting available feature subsets for prediction models. Feature selection serves to eliminate irrelevant or redundant features, resulting in a reduced feature set that can enhance model accuracy and decrease computation time. When dealing with limited data sets, feature selection can improve the generalization ability of machine learning models and mitigate overfitting occurrences. The Random Frog algorithm, originally introduced for gene selection, is a reversible jump Markov chain Monte Carlo (MCMC)-like algorithm Yun et al. (2013). This algorithm was used for feature selection. The process of feature selection includes feature subset search, feature subset evaluation and feature subset verification. Furthermore, choosing an appropriate stopping criterion can not only optimize the feature selection process but also reduce the overall selection time. The core idea of the random frog leaping method is to randomly select feature subsets. In this study, the performance evaluation and ranking of these subsets were conducted using the PLS-DA combined with cross-validation method. The outcomes of the feature selection were utilized to create a feature subset that will be employed for subsequent model classification.

### 2.6 Bruise classification algorithm

The pears with three degrees of bruising were classified. For each degree of bruising, the data set was randomly divided into training set and test set according to the ratio of 7:3.

This study developed three classification methods. LS-SVM is a variant of the standard Support Vector Machine (SVM). Unlike the latter, LS-SVM obtains the final decision function by solving linear equations instead of quadratic programming problems. As a result, it exhibits excellent generalization performance and requires lower computational cost (Suykens and Vandewalle, 1999). In this study, the radial basis function (RBF) kernel function was applied to the calculation of the LS-SVM, and the regularization parameters of the LS-SVM model were determined by ten-fold cross-validation. The purpose was to identify the parameter values that yield the best performance on the given dataset. PLS-DA is a supervised classification method, which was developed using the Partial Least Squares (PLS) algorithm initially designed for multivariate calibration (Wold et al., 2001). When employing the PLS-DA model for classification, it is crucial to ascertain the optimal number of latent variables for modeling. In this study, the number of latent variables in the PLS-DA model was determined based on the criterion of the smallest prediction error observed in the leave-one-out cross-validation. KNN is a widely employed machine learning algorithm for tackling supervised classification tasks. It functions by calculating the distance between various feature vectors and employs cross-validation to determine the most suitable value of K.

To address the variability introduced by data division, each of the aforementioned training instances is replicated 30 times. Each bruise degree and the overall samples were then modeled independently. The training was conducted using two distinct image inputs (AC and RT). Subsequently, a fair comparison was made between the outcomes obtained from the different image inputs and the three classifiers.

Three commonly used metrics are employed to assess the effectiveness of various classification models. These metrics include True Positive (TP), True Negative (TN), and Overall Accuracy (ACC). The TP and TN rates are computed as the ratios of accurately classified bruised and sound samples, respectively, to the total samples in their respective categories. ACC represents the proportion of all correctly classified samples to the total number of test samples. The aforementioned performance indicators are derived from the average values computed across thirty randomly partitioned datasets utilized for modeling.

The image preprocessing, feature extraction, and model training procedures were carried out using Matlab R2017a (The Mathworks, Inc., Natick, MA, USA).

## 3 Results and discussion

### 3.1 Performance of bruising detection based on different spatial frequencies

**Figure 3:**
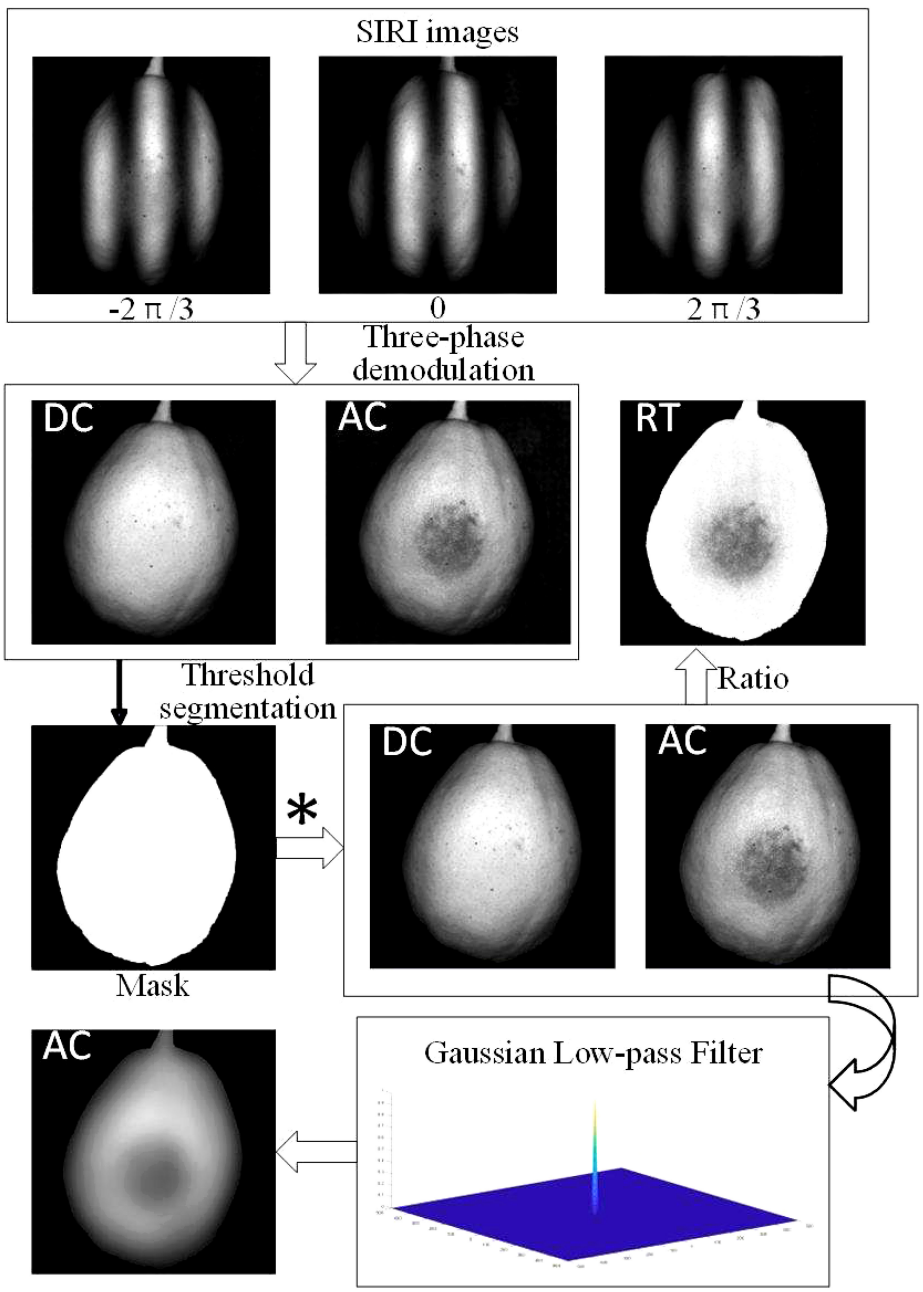
Three-Phase Demodulation and image processing. * Represents the dot product of Mask and DC or AC images.

**Figure 3** shows the basic image processing, including three-phase demodulation, background segmentation and frequency domain filtering. Using the three-phase demodulation method, the collected three SI images can be demodulated to obtain AC images and DC images. Background segmentation mainly used the threshold method to generate the pear area mask to remove the influence of the background on the bruise detection. Here, the DC image was used as a reference, and the mask was generated by manual threshold segmentation. The threshold is manually adjusted in a small increment to obtain the appropriate value, and the morphological operation is supplemented to generate the appropriate mask. Since the detection environment is stable, the value is used to generate a mask for all samples to segment the pear area from the image background. In addition, a Gaussian low-pass filter was used to denoise the AC images and enhance the bruise detection effect of the AC images. The processed AC images were used for the next step of image processing and classification.

**Figure 4:**
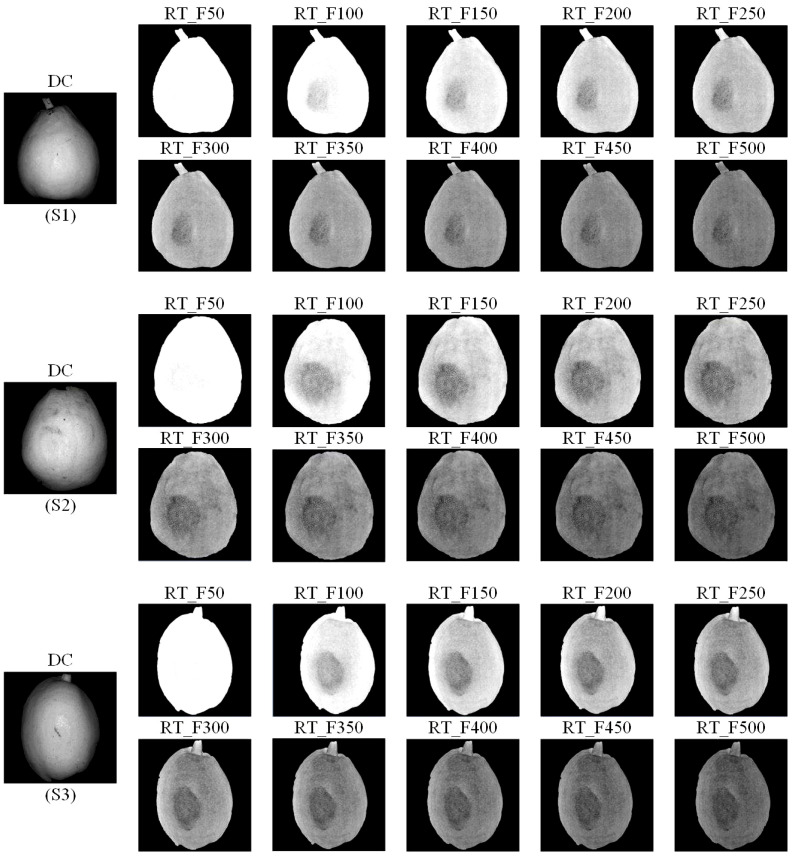
RT images and DC images for the mild (S1), moderate (S2) and severe bruised (S3) ‘Korla’ pears at the spatial frequencies of 50, 100, 150, 200, 250, 300, 350, 400, 450 and 500 cycles m^-1^, respectively.

**Figure 4** displays the DC and RT images of three degrees of bruising (S1, S2, and S3) captured at different spatial frequencies. It should be noted that each spatial frequency produces a DC image, but all DC images remain basically the same. Upon visual inspection, it is observed that except for the spatial frequency of 50 cycles m^-1^, RT images at different spatial frequencies can effectively identify the subcutaneous bruising area of pears, while the DC image, equivalent to the image under uniform illumination, does not show hidden bruises. In addition, the RT image led to a more uniform image background. Due to the curvature of the pear surface, the RT image has a positive effect on the correction of intensity distortions, which can greatly eliminate the influence of uneven illumination, while the DC image obviously shows a darker background edge. As the spatial frequency of the SI increases, the overall brightness of the RT image decreases. At higher spatial frequencies, as the overall brightness decreases, the bruise contrast decreases significantly. The darkening of RT images at high spatial frequencies can be attributed to the characteristics of SI. The SI attenuation rate at high spatial frequency is high, resulting in signal attenuation, so the AC image will be darkened. The brightness of the DC image at different frequencies does not change significantly, so the ratio image eventually darkens, as shown in Equation (4). In general, RT images at all frequencies except the lowest frequency achieved consistently good performance in detecting different fresh bruises on pears.

To further quantify the distinguishability of subcutaneous bruises in pears, the CI values were calculated for different bruise degrees at various spatial frequencies, as presented in **Table 1**. The table reveals a consistent pattern across different degrees of bruising: as the spatial frequency increases, the CI initially rises, reaching a peak at a certain frequency, and then gradually decreases. Indeed, except for the CI at spatial frequency of 50 cycles m^-1^, bruises at low spatial frequencies are more distinguishable, which is in line with the visual observations.

**Table 1:** Contrast indexes (CIs) obtained under for different spatial frequencies (cycles m^-1^) three bruise degrees.

Bruise degree	50	100	150	200	250	300	350	400	450	500
S1	0.205	0.486	0.501	0.478	0.438	0.400	0.359	0.312	0.275	0.235
S2	0.382	0.611	0.625	0.536	0.486	0.433	0.384	0.343	0.309	0.276
S3	0.340	0.592	0.544	0.458	0.393	0.346	0.309	0.280	0.251	0.231

Among them, S1 and S2 samples achieve the maximum CI at the spatial frequency of 150 cycles m^-1^, whereas S3 samples reaches its highest CI at 100 cycles m^-1^. Considering that mild bruises are more difficult to be detected, it is necessary to focus on the detectability of bruises in S1 and S2 samples. Moreover, it can be seen from the table that the CI values at 100 and 150 cycle m^-1^ in S3 are still at a high level. Hence, this study selected 150 cycle m^-1^ as the final spatial frequency for the subsequent bruise detection of all samples.

### 3.2 Image demodulation results

**Figure 5:**
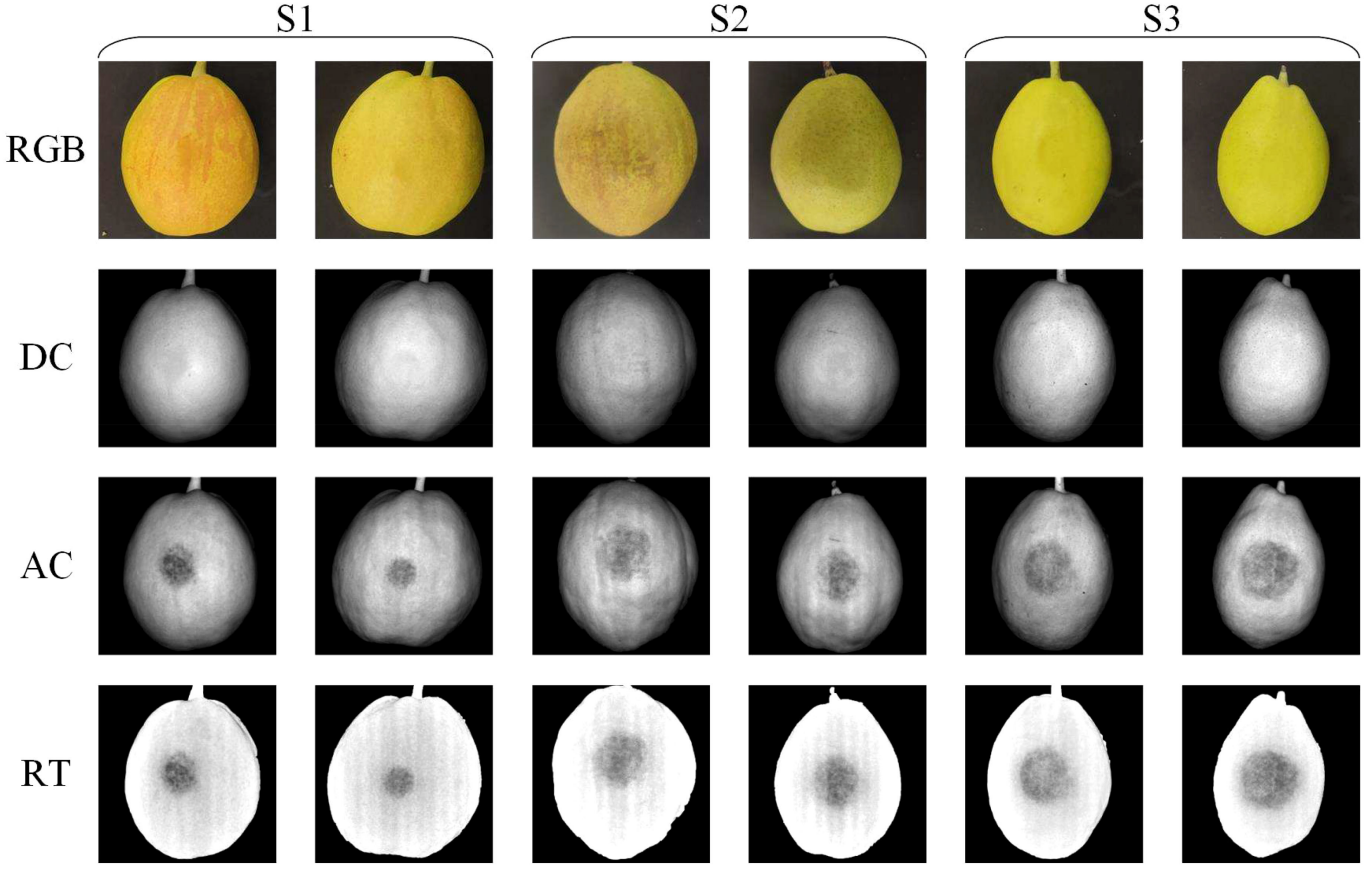
Typical RGB, DC, AC, RT images of mild (S1), moderate (S2) and severe (S3) bruise of ‘Korla’ pears.

**Figure 5** shows typical samples of three different degrees of bruising, all of which were detected immediately after the bruising occurred. These pears underwent varying degrees of bruising when exposed to different levels of static pressure. With naked eyes observation, bruises on pears are readily discernible in AC and RT images, whereas they are almost imperceptible in RGB and DC images. The bruised area appears as a darker region in the image. Nevertheless, it is impractical to determine the extent of bruising by relying solely on the grayscale values in this region. This limitation arises from the lack of discernible differences in intensity among the three distinct levels of bruising, particularly in RT image. The RT image clearly demonstrates effective image enhancement achieved by the ratio of AC to DC image. The contrast in the RT image is noticeably higher compared to the AC image, and it successfully eliminates artifacts resulting from the pear’s surface color and irregular shape.

It can be seen from the **Figure 5** that pears at two maturity stages (green maturation period and yellow maturation period) can obtain good detection results. In addition, although some pears have red stripes, they have no effect on the final detection results including DC, AC and RT images. However, the irregular shape of pears does affect the detection results, mainly in DC and AC images, while RT images completely eliminate this negative influence.

### 3.3 Bruise classification

**Figure 6:**
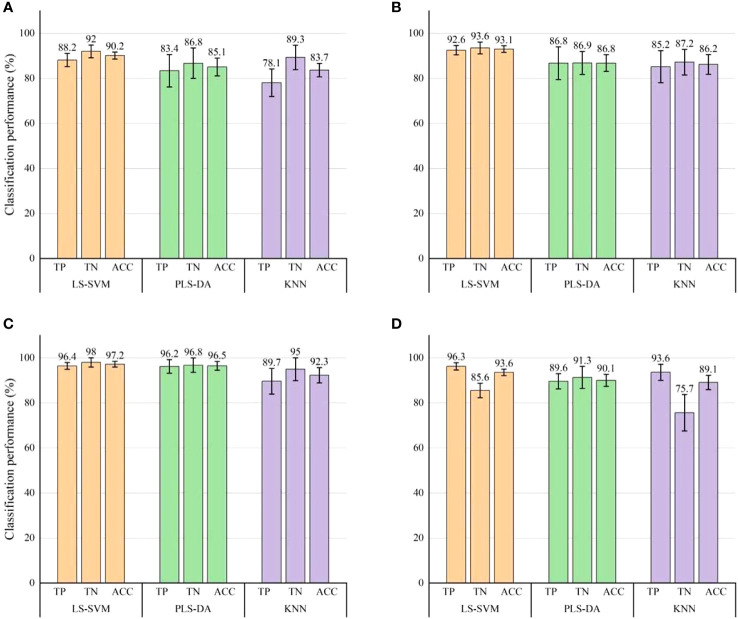
Classification results for bruise detection by using three classification models with AC images. **(A)** Classification results of mild bruises (S1). **(B)** Classification results of moderate bruises (S2). **(C)** Classification results of severe bruises (S3). **(D)** Overall classification results of the three levels of bruising. Error bars on the chart signifies the corresponding standard errors of the evaluation index derived from 30 modeling instances.

**Figure 7:**
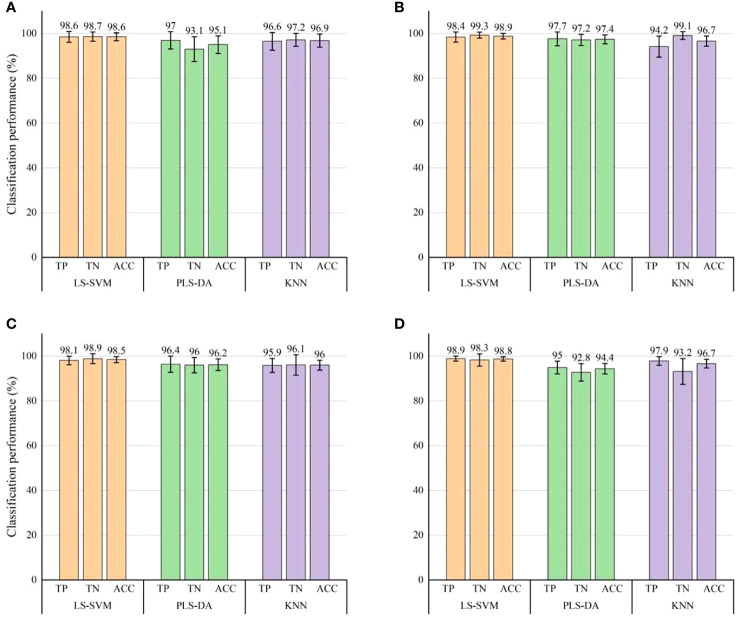
Classification results for bruise detection by using three classification models with RT images. **(A)** Classification results of mild bruises (S1). **(B)** Classification results of moderate bruises (S2). **(C)** Classification results of severe bruises (S3). **(D)** Overall classification results of the three levels of bruising. Error bars on the chart signifies the corresponding standard errors of the evaluation index derived from 30 modeling instances.

The classification outcomes of three classification models (LS-SVM, PLS-DA and KNN) when AC and RT images were employed as inputs for independent data were shown in **Figures 6**, **7**, respectively. The diagrams illustrated that it was viable to employ visible LED SIRI technique to immediately detect the bruising on ‘Korla’ pears, resulting in a commendable level of detection accuracy. The detection accuracy of RT images under each classification model surpasses that of AC images, aligning with the observations made by visual inspection. The LS-SVM model exhibits both the highest detection accuracy and the greatest model stability. When compared to the PLS-DA and KNN models, LS-SVM demonstrates superior detection outcomes across three bruise severity levels and two image inputs. When AC images were used as input, the classification accuracy and stability of the LS-SVM model elevate as the degree of pear bruising. Notably, an overall classification accuracy exceeding 90% can still be achieved in the identification of mild bruising. From the perspective of ACC, the classification accuracy of LS-SVM, PLS-DA and KNN models increased with the increase of pear bruise degree. Among them, the accuracy of PLS-DA in detecting samples with severe bruise degree was close to that of LS-SVM model, but its stability was still not as good as the latter. The KNN model also achieved 92.3% ACC, but its stability is far less than the former two. In actual production, the degree of bruising of pears is not the same, which is related to the environment of pears in production and transportation. Therefore, the overall detection accuracy of different degrees of bruising may be more in line with the actual situation. Although the LS-SVM model achieves high accuracy and stability in the detection of samples with a single degree of bruising, it’s not very outstanding in the detection of bruises in mixed samples with three degrees of bruising due to only 85.6% of sound fruit recognition accuracy. Therefore, AC images may not be suitable for bruise detection of pears in commercial production.

When RT images were used as input, the three classification models show excellent performance in bruise detection accuracy, and were superior to AC images in terms of detection accuracy and model stability. Moreover, according to the error bar, the stability of the LS-SVM model is still higher than that of the other two models. For individual and combined samples with different degrees of bruising, the three evaluation indexes (TP, TN and ACC) of the LS-SVM model all exceeded 98%. Notably, when the classification model was employed for identifying bruises in RT images, it consistently maintains a high level of accuracy in detecting bruises of varying severities, with little fluctuation. This shows that compared with AC images, the detection effect of RT images is less correlated with the degree of pear bruising. The detection accuracy of RT images in each degree of bruise was greater than the best result of AC images in detecting bruises (severe bruises). Therefore, it is a better choice to use RT images as the basis for pear bruising detection. Especially, the detection performance is still very good when the RT image performs mixed detection of pears with different bruising degrees. Hence, RT image was proved to be a more favorable option for detecting bruises on pears with varying degrees. It is feasible to use RT images for bruising detection of ‘Korla’ pears in practice.

**Figure 8:**
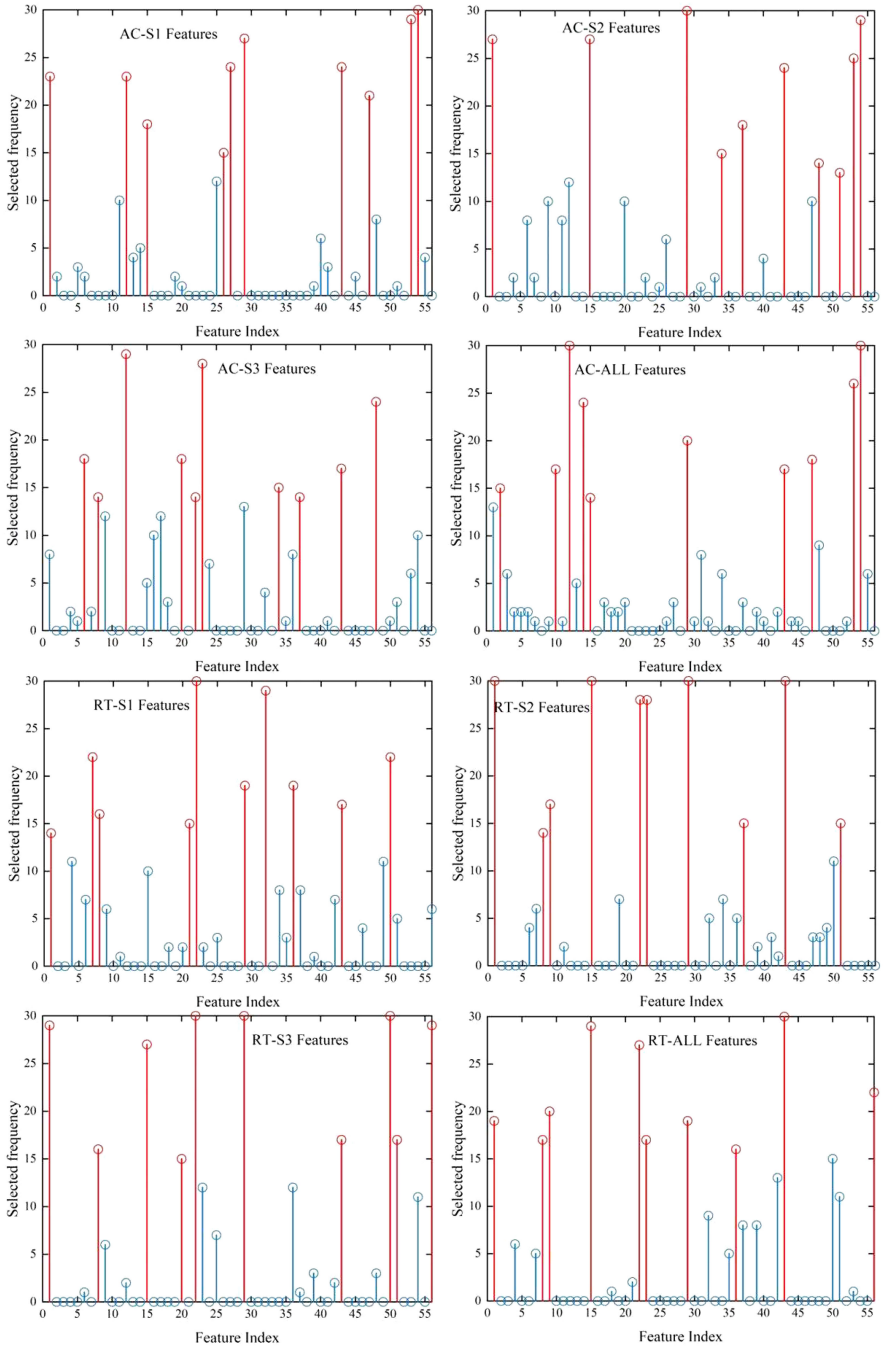
The feature selection results of independent data set and mixed data set of AC image and RT image with mild (S1), moderate (S2) and severe (S3) bruises. The first ten most discriminative features are selected by the random frog feature selection algorithm, and the frequency of each feature selected when the data set is randomly divided for thirty times is counted. The top ten features according to selected frequency are highlighted in red.

In feature selection, different feature subsets will be generated according to the different division of sample sets. In this study, based on the random frog feature selection algorithm, the optimal ten features were selected as feature subsets. With the division of each data set, the number of times each feature is selected as a feature subset is counted, as shown in **Figure 8**. Ten features with the most selected times are selected to establish a new feature subset. It can be seen from the figure that the feature subsets of different degrees of bruising are not the same, and the feature subsets of AC images and RT images are also quite different. Among them, the feature subsets of AC images with different degrees of bruising are quite different, and there are few common features, while there are many common features for RT images, which further proves that the stability of RT image for detection of bruising is higher than that of AC image. In addition, for the mixed data sets of three bruising degrees, the feature subset of the AC image only contains the most frequently selected features in the independent data sets of different bruising degrees, while the RT image contains many common features, which indicates that it is easier to obtain the best subset of features from the feature set of the RT image to achieve the highest classification accuracy. From the perspective of detection accuracy, the accuracy of the classification model with AC image as input will increase with the increase of the degree of bruising, while the classification model with RT image as input has little difference in accuracy, which is consistent with the results of feature selection.

**Table 2** summarizes the classification accuracy of bruised pears by three kinds of models established based on ten features. These features were selected based on the above highest frequency. It can be noted that different models have varying classification accuracy for inputs with different degrees of bruising and AC/RT images. The classification accuracy of the model with RT image as input is still higher than that of AC image regardless of type of model, and the LS-SVM model is the optimal classification model. The overall detection accuracies of slight, moderate, severe and mixed degree of bruising were 99%, 98.11%, 98.44% and 98.64%, respectively. By using the feature subset with the highest frequency, the LS-SVM model improved the effect of detecting mild bruises when RT image was used as input. Further examining the ten selected texture features, it is found that they mainly come from the Angular Second Momen, sum entropy, entropy, and maximum correlation coefficient in different directions, indicating they, combined with RT image, are important for detecting ‘Korla’ pear bruises.

**Table 2:** The classification accuracy (%) of bruised pears by three kinds of models established based on ten features with the highest frequency of selection.

Input	Degree	LS-SVM			PLS-DA			KNN		
		TP	TN	ACC	TP	TN	ACC	TP	TN	ACC
AC	S1	89.33	92.33	90.83	84.78	88.78	86.78	77.33	89.89	83.61
	S2	92.22	94.00	93.11	85.78	89.22	87.50	85.78	90.89	88.33
	S3	96.11	98.56	97.33	94.56	90.00	92.28	89.67	96.44	93.06
	ALL	96.59	88.00	94.44	90.04	94.33	91.11	93.41	79.67	89.97
RT	S1	99.00	99.00	99.00	93.44	92.56	93.00	95.67	97.44	96.56
	S2	97.33	98.89	98.11	88.44	93.56	91.00	98.44	98.44	98.44
	S3	98.00	98.89	98.44	99.44	98.89	99.17	96.44	95.78	96.11
	ALL	99.11	97.22	98.64	92.26	92.89	92.42	96.78	85.44	93.94

ALL refers to a collection of mild (S1), moderate (S2) and severe (S3) bruising samples.

## 4 Conclusion

This study successfully demonstrated the feasibility of low-cost visible-LED SIRI technique for the early detection of varying degrees of subcutaneous bruising in ‘Korla’ pears. The 150 cycles m^-1^ was determined as the optimal structural illumination spatial frequency. For detection of three degrees of bruised pears, RT and AC images were significantly superior to DC images, and RT image was best due to the ability of enhanced image contrast and brightness unevenness correction. Texture features can serve as important features for classifying bruised and sound pears and random frog was an effective texture feature optimization algorithm. Among three types of texture feature models (LS-SVM, PLS-DA and KNN models), the LS-SVM model exhibited superior detection performance with the highest detection accuracy and stability, regardless of single bruising degree classification or mixed bruising degree classification. The LS-SVM model established using ten appropriate features extracted from RT images achieved classification accuracies of 98.6%, 98.9%, 98.5%, and 98.8% for mild, moderate, severe and mixed bruises, respectively, indicating the outstanding ability of the proposed methodology in detecting the bruising of pear fruit in this study. Subsequent study should improve the hardware system and algorithms so that this low-cost SIRI technique can be implemented for online detection of pear bruising. Furthermore, the capacity for the early bruising detection of other thin-skinned fruit (e.g. apple and peach) should be also assessed to expand the application of this technology.

## Data availability statement

The raw data supporting the conclusions of this article will be made available by the authors, without undue reservation.

## Author contributions

MM: Data curation, Methodology, Writing – original draft. ZC: Writing – review & editing, Validation. XZ: Writing – review & editing. CS: Writing – review & editing. JZ: Writing – review & editing. HP: Funding acquisition, Writing – review & editing. JL: Funding acquisition, Writing – review & editing, Supervision. RS: Writing – review & editing, Validation. WZ: Writing – review & editing, Validation.
